# A viral circular RNA in Kaposi’s sarcoma-associated herpesvirus modulates viral and host gene expression during latent and lytic replication

**DOI:** 10.37349/etat.2025.1002320

**Published:** 2025-05-29

**Authors:** Soleil Torres, Vaibhav Jain, Daniel Stribling, Lauren A. Gay, Muhammed Naeem, Melody Baddoo, Erik K. Flemington, Scott A. Tibbetts, Rolf Renne

**Affiliations:** Institute of Oncology Research, USI, Switzerland; IRCCS Istituto Romagnolo per lo Studio dei Tumori (IRST) “Dino Amadori”, Italy; ^1^Department of Molecular Genetics and Microbiology, University of Florida, Gainesville, FL 32601, USA; ^2^UF Genetics Institute, University of Florida, Gainesville, FL 32601, USA; ^3^Department of Pathology and Laboratory Medicine, Tulane University School of Medicine, New Orleans, LA 70112, USA; ^4^UF Health Cancer Center, University of Florida, Gainesville, FL 32601, USA

**Keywords:** Circular RNA, Kaposi’s sarcoma-associated herpesvirus (KSHV), noncoding RNA, cancer

## Abstract

**Aim:**

Circular RNA (circRNA) is a class of noncoding, single-stranded RNA generated by backsplicing, a process where the 5’ and 3’ ends of an RNA are covalently joined. Virally encoded circRNAs have been identified in several members of Gammaherpesvirinae, including Kaposi’s sarcoma-associated herpesvirus (KSHV). In KSHV, the viral interferon regulatory factor 4 (vIRF4) region produces two isoforms of circRNA (circ-vIRF4) that are detectable during latency and reactivation. Given the growing literature implicating circRNA in human diseases, a role may exist for circ-vIRF4 in the development of KSHV malignancies. Therefore, the aim of this study is to characterize the function of vIRF4 circRNAs.

**Methods:**

A KSHV mutant (Δcirc-vIRF4) was generated in the BAC16 bacmid and transfected into 293T and iSLK cells. Expression of circRNA after mutagenesis was assessed by qualitative and quantitative PCR. Host and viral gene expression in iSLK cells during both viral latency and reactivation were also assessed by RNA-seq.

**Results:**

RT-PCR of Δcirc-vIRF4-infected iSLK cells demonstrated no expression of wild-type (WT) isoforms, but PCR cloning showed that alternative backsplice sites were used to express novel vIRF4 circRNAs, where the most prominent isoform was a 1,020 nt isoform. RNA-seq analyses comparing WT- and Δcirc-vIRF4-infected iSLK cells demonstrated significant differential expression of both host and viral genes during both phases of the viral life cycle. Gene ontology analyses returned terms related to cell adhesion, proliferation, and migration for both datasets, as well as kinase signaling and apoptosis for the lytic dataset.

**Conclusions:**

These results show that KSHV can switch to an alternative backsplice site for vIRF4 circRNA production in the absence of a canonical splice site and that circ-vIRF4 contributes to the regulation of both host and viral gene expression through an unknown mechanism.

## Introduction

Kaposi’s sarcoma-associated herpesvirus (KSHV) is a human oncovirus that causes several cancers in humans, including Kaposi’s sarcoma (KS) [1], multicentric Castleman’s disease (MCD) [2], and primary effusion lymphoma (PEL) [3]. KSHV infects endothelial and B cells, establishing lifelong infections via the creation of a latency reservoir. KSHV is a member of the gammaherpesvirus family, which also includes Epstein Barr virus (EBV) and murine gammaherpesvirus 68 (MHV68). It is a double-stranded DNA virus with a genome size of ~160 kb, of which ~140 kb is a long unique coding region that encodes roughly 80 ORFs [4]. In addition to proteins, KSHV encodes a variety of noncoding RNAs. A cluster of twelve miRNA genes is located within the KSHV latency-associated region (KLAR) [5–8]. Viral long noncoding RNAs (lncRNAs) are also expressed, including a 1.1 kb polyadenylated nuclear RNA (PAN) transcript [9] and a 10.1 kb antisense-to-latency transcript (ALT) [10]. KSHV PAN has been found to participate in processes such as viral transcriptional regulation and chromatin remodeling [11], while KSHV miRNAs have been shown to affect processes such as cell proliferation, migration, and angiogenesis [12]. In order to understand how KSHV infection can lead to tumorigenesis, it is essential to study the interactions that coding and noncoding viral transcripts have with each other and the host cellular environment. Currently, there is no cure for KSHV infection or its associated malignancies.

Circular RNA (circRNA) has been established recently as a new class of noncoding RNA [13]. Formation of a circRNA occurs when an upstream 5’ splice acceptor and a downstream 3’ splice donor become covalently bound together in a process known as backsplicing. This closed circle structure lacks both a poly(A) tail and a 7-methylguanosine (m^7^G) 5’ cap, as well as provides resistance to degradation by exonucleases, such as RNase R. The covalent joining of the 3’ end back to the 5’ end leads to the formation of a backsplice junction, a unique feature that distinguishes circRNA from linear RNA. Although research on this class of RNA has exploded recently, no general function has yet been described for circRNA. However, a range of functions has since been identified, including miRNA sponging [14, 15], regulation of parental gene expression [16], competition with linear RNA for splicing [17], interaction with RNA binding proteins [18], participation in innate immunity [19], and involvement in large regulatory networks with other noncoding RNAs [20]. Additionally, while circRNAs are generally regarded as a type of noncoding RNA, circRNAs have been observed to undergo translation [21].

**Figure 1:**
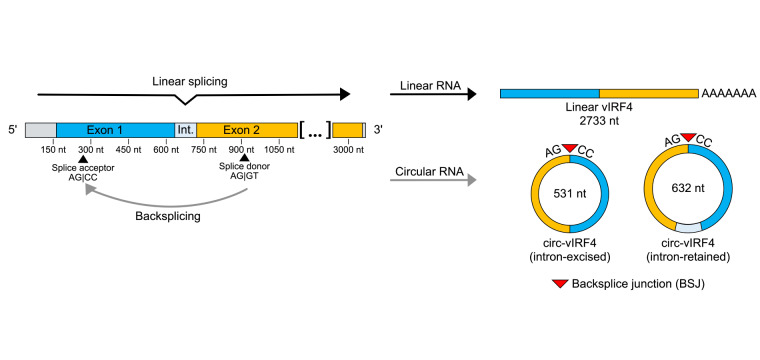
**Visualization of splicing patterns in the vIRF4 locus.** Forward splicing yields a linear, polyadenylated transcript of 2,733 nt that undergoes protein translation. Backsplicing of a splice donor site in exon 2 to a splice acceptor site in exon 1 yields two isoforms of circ-vIRF4: an intron-retained circRNA of 632 nt and an intron-excised circRNA of 531 nt. The covalent bonding from the downstream exon back to the upstream exon results in the creation of the backsplice junction, a feature unique to circRNA. circRNA: circular RNA; vIRF4: viral interferon regulatory factor 4

Several virally encoded circRNAs have been identified in KSHV and EBV via RNase R sequencing. An abundance of viral circRNAs was detected in EBV and led to a description of the EBV circRNAome [22]. In KSHV, circRNAs were detected primarily from two regions: the K10 region, which encodes for viral interferon regulatory factor 4 (vIRF4), and the PAN region [23–25]. vIRF4 is a lytic gene whose mature mRNA comprises of two exons and gives rise to a protein of 911 amino acids. In addition to mRNA, two predominant isoforms of circRNA (circ-vIRF4) are expressed from vIRF4: an intron-retained form of 632 nucleotides and an intron-excised form of 531 nucleotides. While circ-vIRF4 contains sequences from both exons, non-canonical splice sites are used in the backsplicing process, so neither isoform of circ-vIRF4 contains the entirety of either exon (Figure 1).

Both circ-vIRF4 isoforms have been detected in a variety of cell culture models for KSHV, including latently infected cells of lymphoid (BCBL-1), endothelial (TIVE), and epithelial (iSLK) origins [25]. Similarly, circ-vIRF4 has also been detected in primary tumor tissue and sera from KS patients [26]. The localization patterns of circ-vIRF4 have been investigated. Nuclear/cytoplasmic fractionation has demonstrated that exonic circ-vIRF4 is localized to both the nucleus and cytoplasm, while the intron-retained form is localized mainly in the nucleus [23, 26]. Likewise, polysome fractionation has shown that circ-vIRF4 does not associate with translation machinery [23]. Since circ-vIRF4 isoforms are distributed to different compartments of the cell and are not translated, this circRNA may play noncoding roles that are specific to the subcellular compartment occupied by a particular isoform. While circ-vIRF4 is derived from a lytic region, expression of both isoforms is readily detectable during both latency and lytic replication. Additionally, its expression has been observed to increase, decrease, or undergo no change, depending on the cell line examined [25, 26], suggesting that circ-vIRF4 expression is not exclusively regulated by mechanisms tied to reactivation. While these prior studies have elucidated the sequence composition, expression patterns, localization, and stability of circ-vIRF4, the function of this circRNA in KSHV infection remains to be characterized.

Noncoding RNAs are recently being recognized for their roles in disease. In particular, circRNAs have been studied in which they were shown to either suppress or contribute to some cancers. Circ-ITCH suppresses bladder cancer development by sponging miR-17 and miR-224, allowing derepression of p21 and PTEN mRNA [27]. In contrast, circβ-catenin promotes liver cancer by encoding a truncated β-catenin protein that stabilizes full-length β-catenin, promoting activation of the Wnt pathway [28]. Cellular circRNAs can also influence treatment outcomes, as exemplified by crVDAC3, whose expression was shown to reduce the efficacy of trastuzumab deruxtecan in treating HER2-low breast cancer [29]. In the case of KSHV, cellular circRNAs have even been shown to be induced upon KSHV infection and exhibit either antiviral or proviral effects [24, 30]. Currently, the role of circ-vIRF4 in KSHV pathogenesis or tumorigenesis is unknown. However, in consideration of its expression in latency, the period during which KSHV malignancies develop, it is possible that circ-vIRF4 participates in processes related to oncogenesis. Viral gene expression is restricted during latency, so transcripts expressed during this time are possible candidates as mediators of oncogenesis. Investigation of circ-vIRF4 will offer insight into the contribution of circRNAs to cancers caused by oncogenic viruses.

In this work, we have generated a circ-vIRF4 mutant virus in the background of BAC16 [31] by introducing a two-base substitution in the backsplice donor site. We found that circRNA expression from the vIRF4 locus persists in Δcirc-vIRF4, identifying at least one novel isoform during viral reactivation. Using RNA-seq, we also identified hundreds of differentially expressed genes in Δcirc-vIRF4-infected iSLK cells during both phases of the viral lifecycle, which suggested roles for circ-vIRF4 in the extracellular matrix (ECM), as well as cell metabolism and possible regulation of other cellular noncoding RNAs.

## Materials and methods

### Cell culture

iSLK and 293T cells were grown and maintained in DMEM (Fisher, MT10013CM) supplemented with 10% FBS (Gibco, 16000044) and 1% PenStrep (Gibco, 15140122). All cell lines were grown at 37˚C in 5% CO_2_.

### Generation of Δcirc-vIRF4 bacmid via two-step Red recombination

Mutagenesis primers were designed in which two bases within the splice donor site of circ-vIRF4 were substituted (AG|GT to TG|GA). This mutation was chosen to disrupt the backsplice donor site while minimizing disturbances to the sequences of the linear vIRF4 transcript and its encoded protein. The forward primer consisted of 40 nucleotides upstream and 20 nucleotides downstream of the mutation site. The reverse primer consisted of 40 nucleotides downstream and 20 nucleotides upstream of the mutation site. Kanamycin positive selection marker sequences were added to the 3’ ends of both primers.

A kanamycin cassette was generated by using a pEP-KanS plasmid in a PCR with the mutagenesis primers. The PCR was performed using Phusion High-Fidelity PCR Master Mix (New England Biolabs, M0531S) with the following reaction conditions: 98˚C for 5 min, (98˚C for 30 sec, 53˚C for 30 sec, 72˚C for 40 sec) for 34 cycles, and 72˚C for 7 min. The PCR yielded a product of ~1.5 kb. The kanamycin cassette was purified and then digested with DpnI (New England Biolabs, R0176S) to remove residual pEP-KanS plasmid.

In the first Red recombination step, 100 ng of the kanamycin cassette was electroporated into electrocompetent BAC16-containing *E. coli* GS1783 [31] with the following parameters: 1,500 V, 25 µF, 200 ohms, and time constant between 3.5–4.5 ms. Colonies were recovered after electroporation and grown overnight on kanamycin (17 µg/mL) selection plates. To verify insertion of the kanamycin cassette, colony PCR was performed using GoTaq Green Master Mix (Promega, M7122) with the following reaction conditions: 98˚C for 10 min, (95˚C for 30 sec, 51˚C for 30 sec, 72˚C for 1 min) for 34 cycles, and 72˚C for 5 min. Colonies that were positive for the kanamycin selection marker were then verified for intact viral terminal repeats by preparing minipreps (Qiagen, 12462) that underwent NheI digestion (New England Biolabs, R3131S) and subsequent pulsed field gel electrophoresis. Clones that displayed intact viral terminal repeats were selected for the second Red recombination step. For removal of the kanamycin positive selection marker, I-SceI expression was induced by the addition of arabinose to a final concentration of 2%. Colonies were grown overnight on plates containing chloramphenicol (17 µg/mL) and arabinose (1%). To verify removal of the selection marker, colonies were grown on replica plates containing either chloramphenicol (17 µg/mL) and arabinose (1%) or kanamycin (50 µg/mL) and arabinose (1%). Colonies that grew in chloramphenicol, but not kanamycin, were selected for NheI digestion and pulsed field gel electrophoresis for verification of intact viral terminal repeats.

Clones from the second Red recombination step that demonstrated intact viral terminal repeats were used as PCR templates to verify the mutation in the splice donor site. PCR was performed using GoTaq Green Master Mix with the following reaction conditions: 98˚C for 10 min, (95˚C for 30 sec, 51˚C for 30 sec, 72˚C for 1 min) for 34 cycles, and 72˚C for 5 min. PCR products were purified and submitted for Sanger sequencing (GENEWIZ).

### Establishment of Δcirc-vIRF4-containing cell lines

One day prior to transfection, iSLK and 293T cells were each seeded into 6-well plates to achieve a density of ~50% on the day of transfection. Minipreps were prepared from both wild-type (WT) BAC16 and Δcirc-vIRF4, then complexed with FuGENE HD (Promega, E2311) in Opti-MEM Reduced Serum Media (Gibco, 31985062). Cells were incubated with the transfection media for four hours. After the incubation, 250 µL of FBS was added to each well. Two days after transfection, hygromycin B (Gibco, 10687010) was used to select for successfully transfected cells. iSLK cells transfected with either WT BAC16 or Δcirc-vIRF4 were selected with increasing concentrations of hygromycin B. iSLK cells were initially selected with 150 µg/mL hygromycin B, then 300 µg/mL, then 600 µg/mL, and finally 1,200 µg/mL. 293T cells transfected with WT BAC16 or Δcirc-vIRF4 were selected with 50 µg/mL and 100 µg/mL, respectively. Cells containing bacmid DNA were identified by green fluorescence. Once cells were grown and mostly green fluorescent, the cells were transferred from 6-well plates to 15 cm plates.

### PCR detection and cloning of circRNA from KSHV vIRF4 region

iSLK WT BAC16 and Δcirc-vIRF4 cells were grown to 70–80% confluency and either kept uninduced or induced with 1 µg/mL doxycycline and 1 mM sodium butyrate (NaB) for 72 hours. 293T cells were similarly grown and either uninduced or induced with 20 ng/mL TPA and 1 µM VPA. RNA was extracted with RNA-Bee (Tel-Test; discontinued) according to the manufacturer’s protocol. RNA was treated with the TURBO DNA-free Kit (Invitrogen, AM1907). RNase R digestion reactions were made in a 30 µL volume consisting of 5 µg of RNA, 20 units of RNase R (abm, E049), 3 µL 10X reaction buffer (abm, E049), 1 µL RiboLock RNase Inhibitor (Thermo Scientific, EO0381), and water. Control RNase R digestion reactions were made by substituting water in place of RNase R enzyme. Reactions were incubated at 37˚C for one hour, purified using the RNA Clean & Concentrator-5 kit (Zymo Research, R1013) according to the manufacturer’s protocol, and eluted in 10 µL of water. For reverse transcription, cDNA was synthesized from either 2 µg of non-RNase R-digested RNA or the entirety of a 5 µg RNase R-digested reaction using the High-Capacity RNA-to-cDNA kit (Applied Biosystems, 4387406). PCR amplification of circRNA was done with divergent primers using Phusion High-Fidelity PCR Master Mix with HF Buffer under the following reaction conditions: 98˚C for 30 sec, (98˚C for 10 sec, 68˚C for 30 sec, 72˚C for 1 min) for 34 cycles, and 72˚C for 5 min. The products of each circRNA PCR reaction were run on a 1% 1X TAE agarose gel at 100 V for one hour. To determine the sequences of the resulting circRNAs, PCR products from the induced WT BAC16 and Δcirc-vIRF4 reactions were purified and incubated with Taq DNA polymerase (New England Biolabs, M0273S) and 1 mM ATP for A-tailing. Then, the purified PCR products were cloned into a pCR4-TOPO vector, and cloning reaction products were used to transform One Shot TOP10 Chemically Competent *E. coli* (Invitrogen, C404003). The resulting colonies were screened via colony PCR for products of different lengths using universal M13 primers and GoTaq Green Master Mix under the following reaction conditions: 98˚C for 10 min, (95˚C for 30 sec, 44˚C for 30 sec, 72˚C for 1 min) for 34 cycles, and 72˚C for 5 min. The screening process was repeated until a sufficient variety of bands was obtained for each sample type. Colony PCR products of different sizes were selected for purification and sent to GENEWIZ for Sanger sequencing.

### qPCR detection of linear and circular vIRF4 RNA

iSLK cells were grown and either uninduced or induced as previously mentioned. Uninfected iSLK cells were grown without induction reagents. Two biological replicates were processed for each sample condition. RNA was extracted, DNase-digested, RNase R-digested (circRNA only), and used for cDNA synthesis as previously mentioned. qPCR was performed in 10 µL reactions using FastStart Essential DNA Green Master (Roche, 06924204001) for 45 cycles. All samples were normalized to uninfected iSLK cells and the GAPDH housekeeping gene. Relative expression was calculated using the ΔΔCt method.

Table 1 provides all primer sequences used in this work.

**Table 1:** List of primers used in BAC16 mutagenesis, circRNA PCR detection, and linear/circular RNA qPCR detection

**Primer**	**Forward sequence**	**Reverse sequence**
BAC16_mutagenesis	CTGCCTGCTCCGTGTGGATACCAGTGAATGAGGGCGCATCTACCTCTGGAATGGGGTCCTCTGGGACGCGAGGATGACGACGATAAGTAGGG	GAGGTAGATGCGCCCTCATTTCCAACGAGGCCTGCGTAACTTGTCGCGTCCCAGAGGACCCCATAACCAATTAACCAATTCTGATTAG
Mutagenesis_verify	AGAGAACAAAGCTACGAGGAG	GGAACCCGCCACGTAAA
circ-vIRF4_WT_PCR	AACCACGGCTACGCGACG	GAATACCAGCCAGGCGGGATA
circ-vIRF4_WT_qPCR	GTGTGGATACCAGTGAATGAGG	CAAATGCATGGTACACCGAATAC
circ-vIRF4_1020_qPCR	GGATTCCTAGCAGCCAGCTGA	GAATACCAGCCAGGCGGGATA
Linear_vIRF4_qPCR	TTTACCGACAGAGCTGGAGCG	GTTCGAGAGCGTAAGAGGGAGAC
GAPDH	CTTTGGTATCGTGGAAGGACTC	GTAGAGGCAGGGATGATGTTC

circRNA: circular RNA

### RNA-Seq library preparation

For all RNA samples, three biological replicates were processed for each sample condition, and RNA quality was assessed on a Bioanalyzer 2100 to obtain the RNA integrity number (RIN), where only samples with a RIN of 7.0 or higher were used.

For viral latency libraries, iSLK WT BAC16 and Δcirc-vIRF4 cells were grown to 70–80% confluency, and RNA was extracted with RNA-Bee (Tel-Test; discontinued) according to the manufacturer’s protocol. The UF ICBR NextGen DNA Sequencing Core performed DNase digestion and RNA-seq library preparation.

For viral reactivation libraries, iSLK WT BAC16 and Δcirc-vIRF4 cells were grown to ~80% confluency and induced with 1 µg/mL doxycycline and 1 mM NaB for 72 hours. RNA was extracted with RNA-Bee according to the manufacturer’s protocol and treated with TURBO DNA-free™ Kit (Invitrogen, AM1907). Then, ribosomal RNA (rRNA) was depleted from 1 µg of total RNA using the NEBNext rRNA Depletion Kit (Human/Mouse/Rat) (New England Biolabs, E6310L), and samples were subsequently quantified via Qubit. RNA-seq libraries were then prepared using NEBNext Ultra II Directional RNA Library Prep Kit for Illumina (New England Biolabs, E7760L).

After library preparation, the quality and quantity of each viral reactivation library were assessed by TapeStation. Paired-end sequencing was performed on the Illumina NovaSeq6000 by the UF ICBR NextGen DNA Sequencing Core using the S4 2 × 150 sequencing format.

### RNA-Seq processing

In addition to the rRNA depletion step during library preparation, 151 bp paired-end RNA-seq reads were filtered of rRNA using SortMeRNA (v4.3.2) using the “sensitive” database (v4.3.4) with additional (RNA45SN2) transcript sequence. The filtered sequencing datasets were analyzed by the Tulane Virus RNA-Seq and Bioinformatics Core (New Orleans, LA). Due to library quality issues, the viral reactivation condition was analyzed with three biological replicates per sample type, while the viral latency condition was analyzed with two biological replicates per sample type. Read quality was assessed using FASTQC (v0.11.7). To generate aligned (BAM) files, fastq reads were aligned to a combined human (GRCh38.p13; Ensembl release 103) and KSHV (HHV8; NC_009333) reference genome using STAR (v.2.5.2a). Abundance estimates of human and KSHV transcripts were generated using Kallisto (v0.46.0). Differential expression testing was performed using DESeq2 (v1.32.0), and gene set enrichment analysis was performed using GSEA (v.3.0). Gene ontology (GO) and pathway analyses were performed with WebGestalt [32] by supplying a list of differentially expressed genes and a reference list of all genes analyzed in the respective RNA-seq analysis.

RNA-seq reads underwent a quality control step prior to analysis to identify cell culture contamination. No contamination from three species of the *Mycoplasma* genus (*M*. *hyorhinis*, *M*. *hominis*, and *M*. *fermentans*) and *Acholeplasma laidlawii* was found in any of the prepared libraries. All cell lines used in the laboratory are also regularly tested for mycoplasma contamination.

## Results

### Generation of ∆circ-vIRF4 bacmid and transfection in cell culture

**Figure 2:**
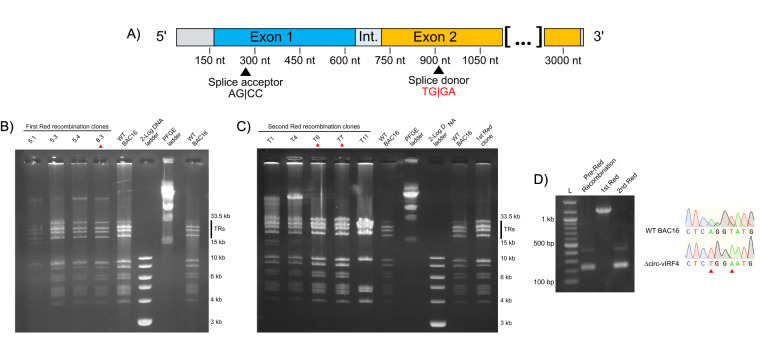
**Generation of a mutant circ-vIRF4 bacmid via 2-step Red recombination and establishment of cell lines.** (**A**) Visualization of the splice donor site mutation. Substituted bases are indicated in red; (**B**) pulsed-field gel electrophoresis (PFGE) of 1st Red recombination. Ideal candidates should display a banding pattern similar to WT BAC16 and have intact terminal repeats (TRs). Clones selected from each recombination step are indicated by a red arrowhead; (**C**) PFGE of 2nd Red recombination. As before, ideal candidates should display a banding pattern similar to WT BAC16 and have intact TRs. Selected clones were chosen for PCR confirmation and sequencing; (**D**) PCR demonstrating insertion and removal of the kanamycin selection marker. Sanger sequencing of the T6 clone from the 2nd Red recombination verified successful incorporation of the splice donor site mutation

To probe the function of circ-vIRF4, a mutation in the backsplice donor site (AG|GT to TG|GA) was generated in BAC16, a bacmid containing the KSHV genome [31], using the two-step Red recombination strategy. Sanger sequencing confirmed that Red recombination successfully produced a marker-less KSHV mutant with the intended base substitutions in the circ-vIRF4 splice donor site (Figures 2A–2D). In addition, 293T and iSLK cell lines containing either WT BAC16 or ∆circ-vIRF4 were established successfully. 293T cells transfected with either bacmid were green fluorescent within 2–3 weeks, while iSLK cells were green fluorescent within four weeks. No deficiency in cell proliferation or abnormal cell morphology was readily apparent in ∆circ-vIRF4 iSLK cells.

### Detection of circRNA from KSHV vIRF4 region

**Figure 3:**
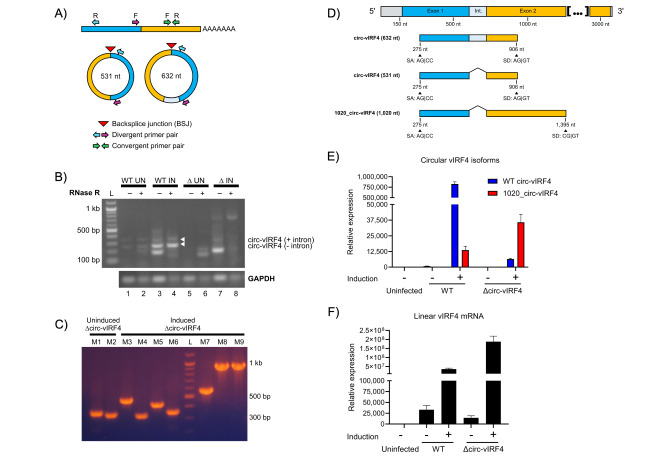
**Analysis of circRNA expression in ∆circ-vIRF4.** (**A**) Convergent versus divergent PCR primers. Convergent, forward-facing PCR primers amplify linear RNA. Divergent, opposite-facing PCR primers amplify circRNA, but not linear RNA. Divergent PCR primers allow for specific amplification of circular isoforms while excluding linear isoforms; (**B**) PCR detection of vIRF4 circRNA in uninduced (UN) and induced (IN) iSLK WT BAC16 and ∆circ-vIRF4 cells. WT isoforms indicated by white arrowheads; (**C**) colony PCR from uninduced and induced iSLK ∆circ-vIRF4 using the same PCR amplicons in Figure 3B; (**D**) splicing graphic of WT and a newly identified vIRF4 circRNA isoform in ∆circ-vIRF4. Sanger sequencing revealed a consistently detectable novel isoform that utilizes a splice donor site located farther downstream of the canonical donor site; (**E**) relative expression of vIRF4 circRNA; (**F**) relative expression of linear vIRF4 mRNA in WT BAC16- and ∆circ-vIRF4-transfected cells. Expression levels were assessed under both uninduced and induced conditions. All samples for circRNA analysis were RNase R-treated, and circRNA was amplified using divergent PCR primers. Ct values were normalized to GAPDH in uninfected iSLK cells, and expression levels were calculated using the 2-∆∆Ct method. A *t*-test (two-sample assuming unequal variances) compared linear vIRF4 expression between WT and ∆circ-vIRF4 in latency and reactivation, returning *p*-values of 0.0112 and 0.0049, respectively. circRNA: circular RNA; vIRF4: viral interferon regulatory factor 4

To assess the effect of mutating the backsplice donor site, detection of circ-vIRF4 using RT-PCR was performed in iSLK cells infected with ∆circ-vIRF4. PCR amplification of circRNA requires the use of opposite-facing (divergent) primers, which specifically amplify circRNA (Figure 3A). Second, RNase R prior to circRNA reverse transcription and PCR is essential for optimal circRNA detection, as artifacts are commonly generated in the absence of previous linear RNA digestion. This is illustrated by lanes 3 and 7, which contain bands amplified from non-RNase R-digested samples that subsequently disappear in lanes 4 and 8, which were RNase R-digested (Figure 3B). In iSLK ∆circ-vIRF4 (lanes 6–9), neither of the two WT isoforms of circ-vIRF4 could be detected under either latency or reactivation conditions. However, multiple other bands were apparent in both of the ∆circ-vIRF4 RNase R-digested lanes (lanes 6 and 8). Cloning and sequencing of these bands (Figure 3C) demonstrated at least one consistently detectable novel circRNA from the vIRF4 region (lanes M8 and M9). Referred to as 1020_circ-vIRF4, this circRNA is 1,020 nucleotides in length, intron-excised, and utilizes the canonical backsplice acceptor site (Figure 3D). However, 1020_circ-vIRF4 uses a backsplice donor site that is located farther downstream within exon 2 and shares sequence similarity with the canonical backsplice donor site. This novel circ-vIRF4 isoform also has a lower level of expression than the WT isoforms. qPCR analysis demonstrated the lower expression level of 1020_circ-vIRF4 in ∆circ-vIRF4 iSLK cells, where its expression during reactivation is approximately 20% of that of the WT isoforms (Figure 3E). Linear vIRF4 expression was also assessed by qPCR and showed that its expression in the mutant was lower in latency, but higher in reactivation in comparison to WT conditions (Figure 3F). Although alteration of linear RNA levels is not ideal in circRNA mutants, we cannot differentiate if this difference is due to the mutation or a possible relationship between the linear and circular forms. Overall, these results demonstrate that while mutation of the canonical donor backsplice site prevents the expression of the two WT circ-vIRF4 isoforms, production of circRNA from the vIRF4 region is not completely abrogated. Rather, alternative backsplice donor sites can be utilized to yield novel isoforms of circ-vIRF4 within this genomic region.

### Differential gene expression analysis of ∆circ-vIRF4

**Figure 4:**
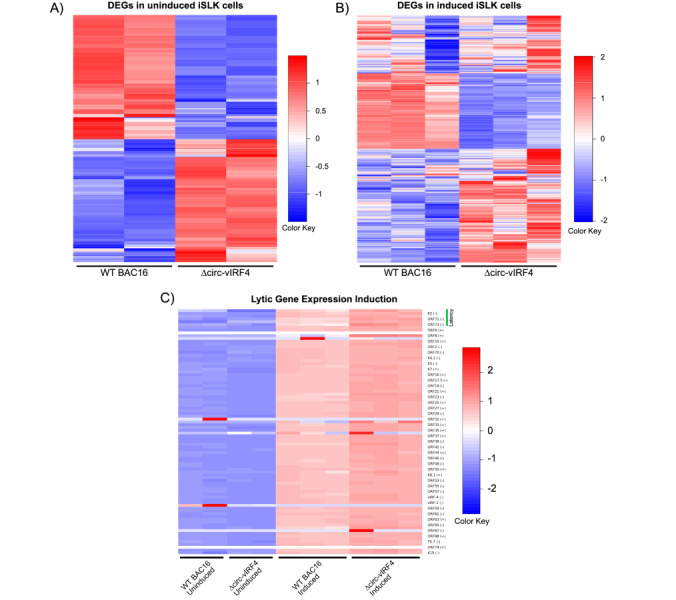
**Gene expression analysis of ∆circ-vIRF4 in iSLK cells.** (**A**) Heat maps of differentially expressed genes for uninduced datasets; (**B**) heat maps of differentially expressed genes for induced datasets; (**C**) heat map of lytic gene expression induction. Blue indicates downregulation and red indicates upregulation. vIRF4: viral interferon regulatory factor 4

**Figure 5:**
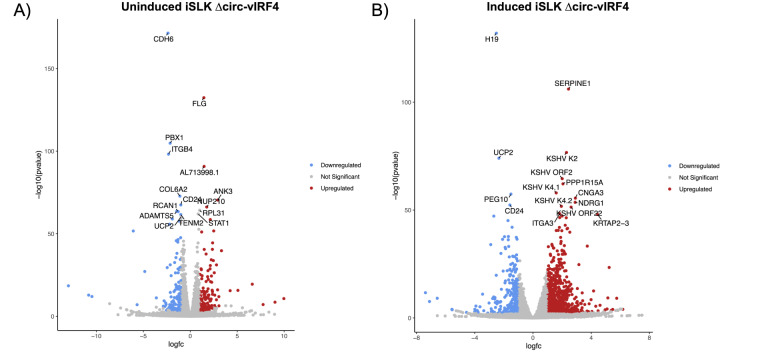
**Volcano plots of differentially expressed genes.** (**A**) Uninduced dataset; (**B**) induced dataset, with the top 15 genes with the most significant *p*-adjusted values labeled. DEGs identified as having log2FC ≥ 1.0 or ≤ –1.0, and a *p*-adjusted value of < 0.05. vIRF4: viral interferon regulatory factor 4

Since a number of cellular circRNAs have been shown to regulate gene expression [33], we wanted to evaluate whether circ-vIRF4 contributes to viral and host gene expression during latent and lytic infection. The RNA-seq analysis demonstrated that both host and viral genes undergo significant differential gene expression under both latency and reactivation conditions in ∆circ-vIRF4. The latency analysis was performed with two biological replicates per WT and ∆circ-vIRF4 condition, while the reactivation analysis was performed with 3 biological replicates per condition. There were 322 and 1,187 differentially expressed host and viral genes with a log2FC ≥ 1.0 or ≥ –1.0, and a *p*-adjusted value of ≤ 0.05 in both latency and reactivation, respectively. Heat maps were generated using all differentially expressed genes for each condition (Figure 4). Volcano plots for each condition display both host and viral genes, where the first fifteen genes with the most significant *p*-adjusted values are labeled (Figures 5A and 5B).

Within the latency dataset, 20 KSHV genes were identified as differentially expressed. Upon exposure to induction reagents for 72 hours, the viral lytic gene expression program was activated in ∆circ-vIRF4 iSLK cells (Figure 4). Within the reactivation dataset, the majority of KSHV genes were upregulated with a log2FC of 2.0–4.0. Some of the more highly upregulated viral genes included ORF8 (log2FC = 5.75) and ORF32 (log2FC = 4.12), both of which are virion-associated proteins. Expression of linear vIRF4 was also assessed in both ∆circ-vIRF4 datasets. Linear vIRF4 expression followed the same pattern of upregulation as the rest of the viral genes in the reactivation dataset (log2FC = 1.9), as did the rest of the vIRFs 1–3 (log2FC of 1.7–2.0). In the latency dataset, however, linear vIRF4 was the only vIRF with differential expression, showing decreased expression (log2FC = –1.712).

**Figure 6:**
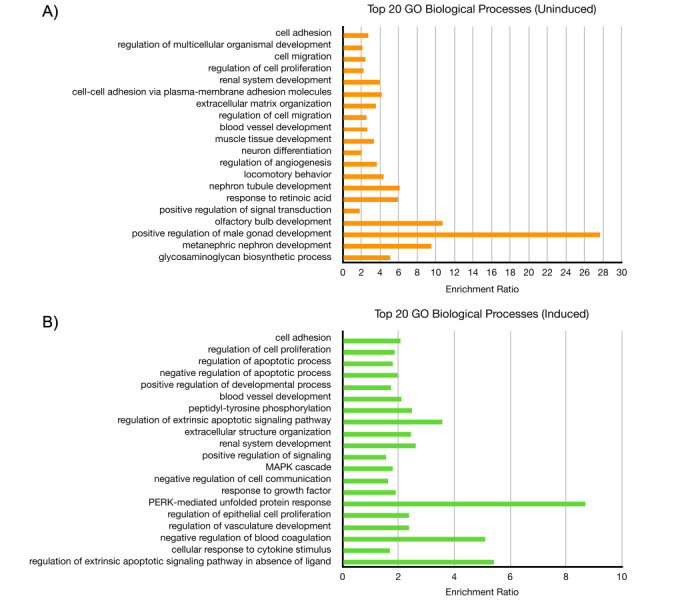
**Gene ontology (GO) analysis of ∆circ-vIRF4 in iSLK cells.** (**A**) The top 20 biological processes from uninduced dataset; (**B**) the top 20 biological processes from induced dataset, as determined by the most significant FDR ≤ 0.05 and listed in descending order. FDR: false discovery rate

As for host genes, the genes with the most significant *p*-adjusted values in both datasets were those related to the ECM, including collagen-, integrin-, and keratin-associated genes. In the reactivation dataset, these included genes such as *SERPINE1*, *KRTAP2-3*, *ITGA3*, and *CDH6*. In the latency dataset, this included genes such as *ITGB4*, *FLG*, *ANK3*, and *COL1A1*. Both datasets also included a few members from the matrix metalloproteinases (MMPs) and a disintegrin and metalloproteinase with thrombospondin motifs (ADAMTSs) families. Consequently, GO analysis of biological processes returned GO terms related to cell adhesion, migration, and proliferation in the latency dataset (Figure 6A). For reactivation, GO terms similarly included processes related to cell adhesion and proliferation, as well as kinase signaling, apoptosis, and unfolded protein response (Figure 6B). Pathway analysis of both datasets using the KEGG database on WebGestalt revealed enrichment for several signaling pathways, including the PI3K-Akt, JAK-STAT, MAPK, NOD-like receptor, and IL-17 signaling pathways.

The latency and reactivation datasets had a handful of differentially expressed host genes in common with *p*-adjusted values ≤ 0.05. Among those genes were *CDH6* (latent log2FC = –2.37; reactivation log2FC = –1.91), *CD24* (latent log2FC = –1.01; reactivation log2FC = –1.59), *PBX1* (latent log2FC = –2.15; reactivation log2FC = –2.20) and *UCP2* (latent log2FC = –0.97; reactivation log2FC = –2.34), all of which were downregulated in both phases. In the latency dataset, *CDH6* was a downregulated (log2FC = –2.38) host gene with the most significant *p*-adjusted value. In the reactivation dataset, *H19* was a host lncRNA that was downregulated (log2FC = –2.52) and had the most significant *p*-adjusted value, while *SERPINE1* was an upregulated protein-coding gene (log2FC = 2.44) with the second most significant *p*-adjusted value. Lastly, in addition to *H19*, other lncRNAs in the reactivation dataset were differentially expressed, including *MALAT1* (log2FC = 1.58) and *GAS5* (log2FC = 1.67). These results indicate that mutation of the canonical backsplice donor site in ∆circ-vIRF4 and its subsequent disruption of WT circ-vIRF4 production have a significant impact on cellular and viral processes.

## Discussion

In this work, a KSHV mutant was generated in which the vIRF4 region harbors a mutation in the canonical splice donor site used for expressing two circ-vIRF4 isoforms. Through the study of ∆circ-vIRF4, insight was given regarding production and possible functions of circ-vIRF4. One novel vIRF4 circRNA was consistently detectable in ∆circ-vIRF4-infected cells, demonstrating that mutation of the backsplice donor site does not preclude expression of circRNA from the vIRF4 locus. In addition, gene expression analysis of ∆circ-vIRF4 revealed differentially expressed host and viral genes during both latency and reactivation, indicating that circ-vIRF4 actively participates in both cycles of the KSHV lifecycle. Because both circ-vIRF4 isoforms are expressed in latency, when the viral episome is transcriptionally restricted and tumorigenesis is known to occur, it is important to investigate how these noncoding RNAs contribute to the development of KSHV malignancies. These clues will lay the groundwork for future studies aimed at further characterization of circ-vIRF4.

RT-PCR of ∆circ-vIRF4 RNA revealed that when a canonical backsplicing site is unavailable, the KSHV vIRF4 locus can generate novel circRNAs using alternative backsplicing sites. The use of alternative splice sites to produce cellular circRNAs has been observed previously. Survival Motor Neuron genes can give rise to nearly 30 unique circRNAs using both canonical and cryptic splice sites located within exons and introns [34]. Likewise, through a computational pipeline designed to detect backsplice junctions from RNA-seq datasets, Zhang et al. [35] observed that alternative backsplicing is common in human circRNA-producing genes. Of note, the sequence of the alternative splice donor site used to express 1020_circ-vIRF4 was similar to the canonical donor site, supporting that there are specific sequence elements within vIRF4 pre-mRNA that guide the production of circ-vIRF4. It also remains to be seen how well novel vIRF4 circRNAs function as suitable replacements for WT circ-vIRF4, if they do at all. Although 1020_circ-vIRF4 retains the full-length nucleotide sequence of the WT isoforms, significant differential gene expression is still observed. One reason for this could be disruption of RNA structures that are necessary for circ-vIRF4 to interact with binding partners. Additionally, the 1020_circ-vIRF4 isoform is expressed at lower levels, so it may not be abundant enough to exert its effects. Regardless, the fact that circRNA is still produced from the vIRF4 region, albeit with significantly reduced levels, despite the unavailability of a canonical splice site, may indicate that there is a need in KSHV infection that is specifically fulfilled by vIRF4-derived circRNA.

The RNA-seq analysis demonstrated that disrupting WT circ-vIRF4 expression affected both host and viral gene expression during both phases of the KSHV lifecycle. According to the GO analysis, cellular processes such as migration, adhesion, and proliferation were most affected in both latency and reactivation. Several types of ECM-related genes, including integrins, collagens, keratins, ADAMS, and MMPs, were identified in the differential expression analysis. This indicates that circ-vIRF4 may have a role in altering the ECM, which could impact the development of a pro-tumorigenic environment. *SERPINE1*, a serine proteinase inhibitor, was an upregulated host gene in the reactivation dataset with a highly significant *p*-adjusted value. *SERPINE1* encodes plasminogen activator inhibitor-1 (PAI-1), a key component of the plasminogen system, which normally functions to modulate the ECM during tissue injury [36]. Within a cancer context, high *SERPINE1* expression is considered an indicator of a poor prognosis in several types of cancer and is hypothesized to function by affecting processes such as cell motility or angiogenesis, a hallmark of KS [37]. In addition, *SERPINE1* has also been shown to act as an interferon-stimulated gene that impedes virion maturation in influenza A infection [38]. By further interrogation of the relationship between circ-vIRF4 and *SERPINE1*, as well as other ECM-associated genes, a clearer role may emerge for how circ-vIRF4 remodels the ECM to accommodate KSHV infection or tumorigenesis.

Another avenue to explore is the ability of circ-vIRF4 to affect cell metabolism. UCP2 is a part of the family of mitochondrial carrier proteins that acts as an intra-mitochondrial metabolite transporter and remodeler of metabolic activity [39]. While the role of UCP2 in cancer is not yet clear, some studies indicate that UCP2 can either promote or suppress tumor development, depending on the stage of the tumor and cell type [40]. As UCP2 was downregulated in both latency and reactivation, there may be a role for circ-vIRF4 in cell metabolism regulation during both phases of the viral lifecycle.

Lastly, a handful of cellular lncRNAs were observed to be differentially expressed during KSHV reactivation, including *H19*, *MALAT1*, and *GAS5*. High *H19* expression has been shown to be a contributor to several human cancers, including bladder, colorectal, and prostate cancers [41]. *GAS5* is a regulator of cell proliferation and apoptosis and known to have reduced expression in a variety of human cancers, including several B-cell cancers [42]. *MALAT1* has been described to promote lung cancer, and its high expression is typically a marker of poor prognosis [43]. This demonstrates the ability of a viral circRNA to affect the expression of cellular lncRNAs known to be consequential to cancer development and progression. Interestingly, both *H19* and *MALAT1* have been identified as targets of a KSHV miRNA [44], suggesting that these cellular lncRNAs could be regulated by more than one class of KSHV-encoded noncoding RNA. Many interesting genes and biological processes were identified by the RNA-seq analyses, but validation via qPCR and circRNA-pulldown studies will be important in elucidating how circ-vIRF4 functions.

In the ∆circ-vIRF4 mutant, we saw no expression of WT isoforms and very little expression of 1020_circ-vIRF4. While there are interesting gene expression changes that allow us to propose a variety of possible roles for circ-vIRF4, it should be noted that we cannot determine with absolute certainty from the current results if the changes in gene expression are the result of the absence of WT isoforms or the presence of 1020_circ-vIRF4. However, this could be elucidated in the future by developing other circ-vIRF4 mutants that are confirmed to lose circRNA expression entirely from the vIRF4 region or by designing siRNAs that knock down validated alternative circ-vIRF4 isoforms prior to creation of sequencing libraries. Additionally, it was observed that the level of linear vIRF4 RNA during latency was altered in the ∆circ-vIRF4 mutant. While it is possible that the mutation could have had an effect on linear RNA production, it is also possible that circ-vIRF4 exerts some control over the expression of mRNA from its parental gene, as is commonly observed in genes in eukaryotic organisms [45].

The results in this work present exciting opportunities to further investigate not only the function of circ-vIRF4 but also its generation. In addition to the vIRF4 locus, KSHV also expresses circRNA from the PAN region; however, these circRNAs vary significantly in size, given that PAN circRNAs do not have consensus backsplice sequences [23]. In contrast, circ-vIRF4 has canonical backsplice sites, and we showed that its WT expression is affected by manipulating at least one splice donor site sequence. While the mechanism of backsplicing is not yet well-understood, splicing factors and repetitive elements have been demonstrated to contribute to cellular circRNA backsplicing [17, 46, 47]. Similarly, circ-vIRF4 expression has been shown to be affected by splicing factors, including the KSHV viral splicing factor, ORF57 [48], and a cellular alternative splicing factor, FUS [49]. Through a better understanding of KSHV circRNA generation, structure, and function, these circRNAs could represent potential therapeutic targets in the treatment of KSHV infection and malignancies. This can be achieved through probing of circRNA-protein interactions, interactions with other classes of RNAs, and circRNA structural features.

The field of circRNA has grown since its recent inception as a new class of noncoding RNAs. Much of the literature has focused mainly on cellular circRNAs, which have been studied in many contexts, including organism development and disease. Due to their unique properties, circRNAs are also being investigated for their potential to serve as therapeutic agents for a variety of human cancers, genetic diseases, and infections [50, 51]. The discovery that some viruses express their own circRNAs adds to the complexity of viral genomes and demonstrates that there is potential for important contributions to be made by noncoding RNAs during viral infections. For example, some EBV-encoded circRNAs have been shown to induce stemness in EBV-associated gastric cancer [52] and facilitate tumor immune escape in nasopharyngeal carcinoma [53]. Studying circ-vIRF4, which is expressed during both latency and lytic replication, in KSHV infection will lead to a better understanding of how viral circRNAs promote infections, and particularly in tumorigenic viruses, how these contributions lead to cancer development.

## Abbreviations

circRNA: circular RNA
EBV: Epstein Barr virus
ECM: extracellular matrix
GO: gene ontology
KS: Kaposi’s sarcoma
KSHV: Kaposi’s sarcoma-associated herpesvirus
lncRNAs: long noncoding RNAs
MMPs: matrix metalloproteinases
RIN: RNA integrity number
rRNA: ribosomal RNA
vIRF4: viral interferon regulatory factor 4
WT: wild-type

## Declarations

### Availability of data and materials

RNA-seq datasets have been submitted to NCBI Gene Expression Omnibus (GEO) and can be accessed under GSE293213.

### Copyright

© The Author(s) 2025.
